# Genomic and Structural Characterization of Kunitz-Type Peptide LmKTT-1a Highlights Diversity and Evolution of Scorpion Potassium Channel Toxins

**DOI:** 10.1371/journal.pone.0060201

**Published:** 2013-04-03

**Authors:** Zongyun Chen, Fan Luo, Jing Feng, Weishan Yang, Danyun Zeng, Ruiming Zhao, Zhijian Cao, Maili Liu, Wenxin Li, Ling Jiang, Yingliang Wu

**Affiliations:** 1 State Key Laboratory of Virology, College of Life Sciences, Wuhan University, Wuhan, P.R. China; 2 Key Laboratory of Magnetic Resonance in Biological Systems, Wuhan Center for Magnetic Resonance, State Key Laboratory of Magnetic Resonance and Atomic and Molecular Physics, Wuhan Institute of Physics and Mathematics, Chinese Academy of Sciences, Wuhan, P.R. China; University of Saskatchewan, Canada

## Abstract

**Background:**

Recently, a new subfamily of long-chain toxins with a Kunitz-type fold was found in scorpion venom glands. Functionally, these toxins inhibit protease activity and block potassium channels. However, the genomic organization and three-dimensional (3-D) structure of this kind of scorpion toxin has not been reported.

**Principal Findings:**

Here, we characterized the genomic organization and 3-D nuclear magnetic resonance structure of the scorpion Kunitz-type toxin, LmKTT-1a, which has a unique cysteine pattern. The *LmKTT-1a* gene contained three exons, which were interrupted by two introns located in the mature peptide region. Despite little similarity to other Kunitz-type toxins and a unique pattern of disulfide bridges, LmKTT-1a possessed a conserved Kunitz-type structural fold with one α-helix and two β-sheets. Comparison of the genomic organization, 3-D structure, and functional data of known toxins from the α-KTx, β-KTx, γ-KTx, and κ-KTx subfamily suggested that scorpion Kunitz-type potassium channel toxins might have evolved from a new ancestor that is completely different from the common ancestor of scorpion toxins with a CSα/β fold. Thus, these analyses provide evidence of a new scorpion potassium channel toxin subfamily, which we have named δ-KTx.

**Conclusions/Significance:**

Our results highlight the genomic, structural, and evolutionary diversity of scorpion potassium channel toxins. These findings may accelerate the design and development of diagnostic and therapeutic peptide agents for human potassium channelopathies.

## Introduction

Over the last 400 million years, scorpions have evolved many peptide toxins that target different potassium channels [1]. Numerous potassium channel toxins have been isolated from scorpions including those identified by proteomic and transcriptome analysis of scorpion venom glands [2]–[4]. These toxins are divided into α-KTx, β-KTx, γ-KTx, and κ-KTx subfamilies based on their similarity [5], [6]. Some of the toxins are specific inhibitors that serve as useful pharmacological tools and candidate drugs that target various potassium channels [7]. Examples include charybdotoxin (ChTX), which is targeted toward Kv1.3 and BKCa channels [8], scyllatoxin (ScyTx), which inhibits SKCa channels [9], maurotoxin (MTX), which is targeted toward IKCa channels [10], and BeKm-1, which inhibits Herg channels [11].

Despite the molecular diversity of scorpion potassium channel toxins, only two structural scaffolds have been found [12]. One is the classical CSα/β fold, which comprises one or two short α-helices connected to a triple-stranded anti-parallel β-sheet stabilized by three or four disulfide bonds. The other is the unique cystine-stabilized α-helix-loop helix (CSα/α) fold, which comprises two α-helices [5]. Recently, a new kind of long-chain scorpion potassium channel toxin was functionally characterized, which has both protease and potassium channel inhibiting properties [4], [13], [14]. Amino acid sequence analyses showed that this kind of scorpion toxin might adopt a unique Kunitz-type fold [15]. However, the three-dimensional (3-D) structures and features of this kind of toxin remain unclear.

In this work, we report the nuclear magnetic resonance (NMR) structure and genomic organization of the scorpion Kunitz-type toxin, LmKTT-1a. The NMR experiments show that LmKTT-1a adopts a conserved Kunitz-type structural fold [16], which is different from other scorpion potassium channel toxins including α-, β-, and γ-potassium toxins (KTxs), which have a CSα/β fold, and κ-KTxs, which have a CSα/α fold [1]. Based on the genomic and functional data, we propose that scorpion Kunitz-type toxins are a new subfamily of potassium channels, which we have named δ-KTx. Our results demonstrate that scorpion potassium channel toxins have greater diversity than previously realized and highlight a new role for convergent evolution of animal toxins.

## Materials and Methods

### Gene Cloning of Representative Scorpion Potassium Channel Toxins

To identify the upstream region of the gene, we amplified genomic DNA using a genome walking kit (Takata, Japan). Net-polymerase chain reaction (PCR) was used to amplify the downstream and 3′ flanking regions of the gene. This method consists of four nested gene-specific primers and two PCR steps. The second PCR product was used for purification and was ligated into the pGEM-T Easy Vector (Promega, USA) for sequencing. *Escherichia coli* JM109 was used for plasmid propagation. Positive clones were sequenced.

### Construction of Expression Vectors

We used the cDNA sequence of LmKTT-1a from a cDNA library of *Lychas mucronatus* venom glands as a template for PCR to generate the fragment. The PCR product was digested with NdeI and BamHI and inserted into expression vector pET-28a. After being confirmed by sequencing, plasmid pET-28a-LmKTT-1a was transformed into *E. coli* Rosetta (DE3) cells for expression. The QuikChange Site-Directed Mutagenesis Kit (Stratagene, USA) was used to generate mutants from the wild-type plasmid, pET-28a-LmKTT-1a, which were verified by DNA sequencing.

### Expression and Purification of LmKTT-1a

To produce ^13^C/^15^N-labeled LmKTT-1a, recombinant cells containing the LmKTT-1a expression plasmid were cultured and induced in M9 medium containing ^15^N-NH_4_Cl as the only nitrogen source and ^13^C-glucose as the only carbon source. The refolding, separation, and identification of LmKTT-1a was performed as previously described [13]. Briefly, the recombinant LmKTT-1a protein was expressed in inclusion bodies and then refolded in vitro at 16°C. The refolded protein was finally purified by high-performance liquid chromatography (HPLC) on a C18 column (10 mm × 250 mm, 5 µm; Elite-HPLC, China). The fraction containing recombinant LmKTT-1a was eluted after 20 to 21 min and was further analyzed by MALDI-TOF-MS (Voyager-DESTR, Applied Biosystems). Unlabeled LmKTT-1a and its mutants were expressed in LB culture according to the same protocol described above.

### Circular Dichroism (CD) Analysis of rLmKTT-1a and its Analogues

The secondary structures of Kunitz-type toxin LmKTT-1a and its mutants were analyzed by CD spectroscopy. All samples were dissolved in water at a concentration of 0.2 mg/ml. Spectra were recorded at 25°C from 250 to 190 nm with a scan rate of 50 nm/min on a Jasco-810 spectropolarimeter. CD spectra data were collected by subtracting the blank spectrum of water and averaging three scans.

### Electrophysiological Recordings

The cDNAs encoding mKv1.1, hKv1.2, and mKv1.3 were generously provided by Professor Stephan Grissmer (University of Ulm, Ulm, Germany) and were subcloned into the pIRES2-EGFP vector (Clontech). Kv1.1, Kv1.2, and Kv1.3 plasmids were then transiently transfected into HEK293 cells. The whole-cell patch clamp was used to measure and record the channel currents according to a previously described procedure [17].

### Serine Protease Inhibitor Activity Assay

The inhibitory activities of LmKTT-1a and its mutants were tested using methods that were previously described [18], [19]. The initial rate of every reaction was monitored continuously at 405 nm for 5 min at 25°C. The inhibitory constant (Ki) of the protease/inhibitor complex was determined by Lineweaver-Burk plots and slope re-plotting.

### Solution Structure Determination of LmKTT-1a

All of the NMR experiments were carried out at 298 K on a Bruker Avance-III 800 MHz spectrometer. The NMR sample contained 1 mM ^13^C/^15^N-labeled LmKTT-1a in 20 mM phosphate buffer with 10% deuterium oxide (D_2_O) (vol/vol) at pH 6.0. The backbone and side chain assignments of LmKTT-1a were determined from the following two- and three- dimensional NMR experiments: ^1^H-^15^N-HSQC, ^1^H-^13^C-HSQC, HNCO, HNCA, HN(CO)CA, HNCACB, CBCACONH, and ^13^C-HCCH-TOCSY. 3-D ^15^N-NOESY-HSQC and ^13^C-NOESY-HSQC spectra were recorded to generate inter-proton distance restraints. All of the spectra were processed with NMRPipe [20], and the assignments were accomplished using Computer-Aided Resonance Assignment [21]. Backbone dihedral angle restraints were derived from proton chemical shifts by TALOS (Torsion Angle Likelihood Obtained from Shift) [22]. The Cyana2.1 [23] and Assisted Model Building with Energy Restraint force field were used to calculate and refine the structures. Three pairs of disulfide bond restraints were added to the distance restraints based on the NOE evidences and position of cysteines from initial structure calculation (Fig. S4). Hydrogen bond restraints were obtained from the hydrogen-deuterium (H-D) exchange experiment after dissolving the lyophilized sample in D_2_O for 8 hours and adding it to the final run of the structure calculation (Fig. S3). The final 20 structures of LmKTT-1a with the lowest energies were assessed by PROCHECK-NMR [24] and graphically analyzed by MOLMOL [25]. It should be noted that recombinant LmKTT-1a contains 79 residues including a 20-residue expression tag at the N-terminus. The intact LmKTT-1a peptide contains 59 residues from Lys1 to Cys59. The protein structure was deposited into the Protein Data Bank (code 2M01) and the BioMagResBank (accession number 18789).

## Results

### Amino Acid Sequence Analysis of LmKTT-1a

**Figure 1:**
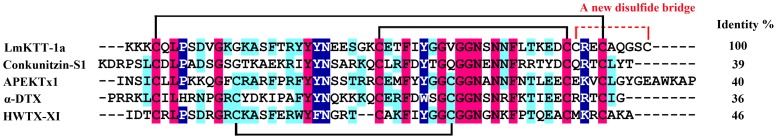
Amino acid sequence alignment of LmKTT-1a with Kunitz-type toxins from other venomous animals. Representative Kunitz-type toxins are LmKTT-1a from scorpion, Conkunitzin-S1 (PDB Code: 1Y62) from conus, APEKTx1 (PDB Code: 1WQK) from sea anemone, α-DTX (PDB Code: 1DTX) from snake, and HWTXI-XI (PDB Code: 2JOT) from spider. The known disulfide bridges are labeled in black lines. The red dotted line suggests a possible new disulfide bridge.

Multiple sequence alignments showed that LmKTT-1a shared little homology with Kunitz-type toxins from other venomous animals, such as HWTX-XI from spider [26], APEKTx1 from sea anemone [27], α-DTX from snake [28], and conkunitzin-S1 from cone snail [29]. In addition, LmKTT-1a possessed a unique cysteine framework. It lacked the normal CysII–CysIV disulfide bridge but contained two cysteine residues at the C-terminus that might generate a new disulfide bridge (Fig. 1).

### Genomic Organization of LmKTT-1a

**Figure 2:**
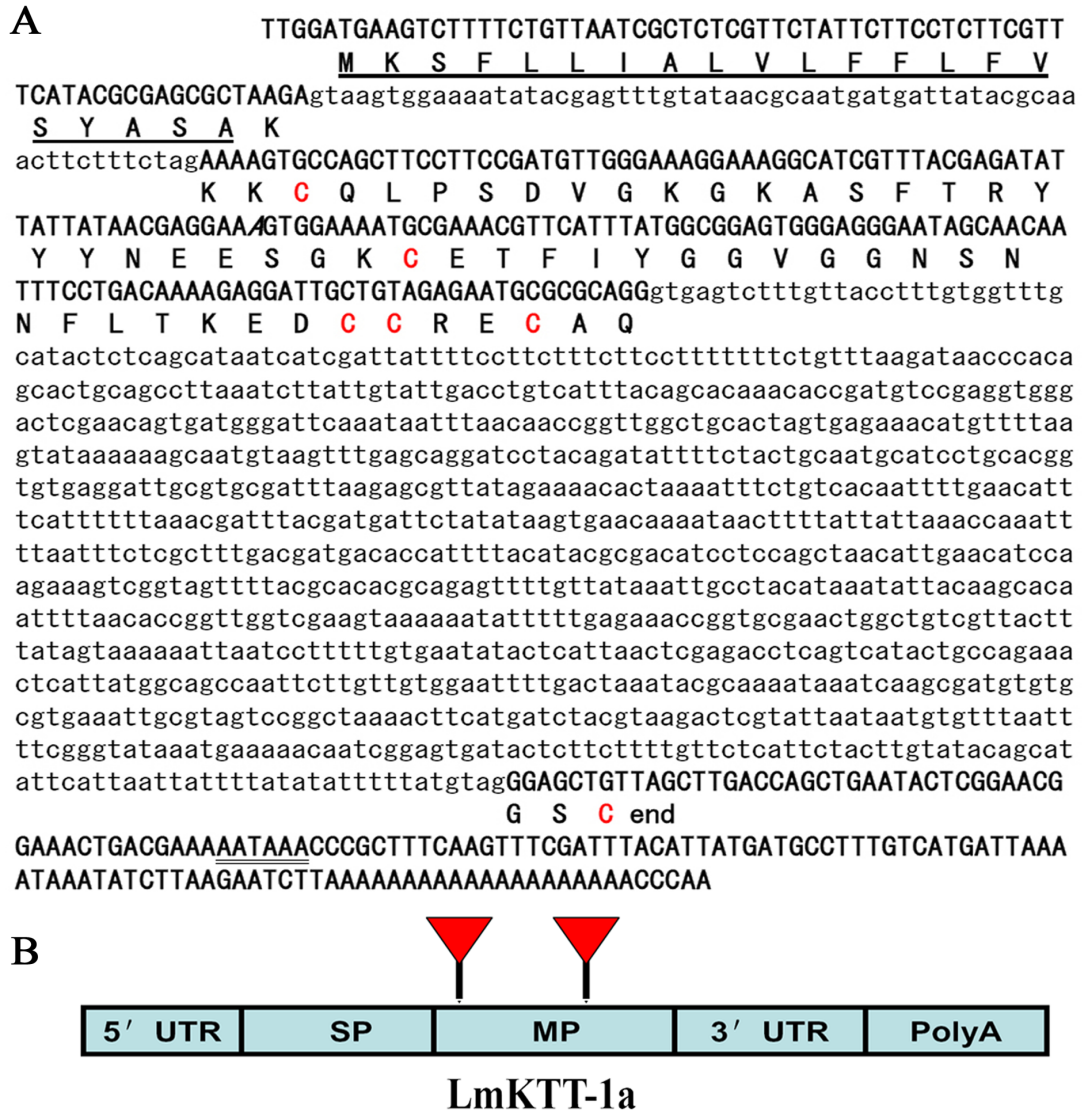
Genomic organization of scorpion Kunitz-type toxin, LmKTT-1a. (A) The *LmKTT-1a* gene. The signal peptide sequence predicted from the nucleotide sequence is underlined. The putative polyadenylation signal (AATAAA) is underlined twice. (B) The gene structure of LmKTT-1a. The signal peptide (SP), mature peptide (MP), 5′-UTR, and 3′-UTR non-coding regions are shown. Introns are designated by triangles.

To determine the gene structure of LmKTT-1a, we first obtained the sequence of genomic DNA (Fig. 2A). A comparison of the LmKTT-1a genomic sequence with the corresponding cDNA sequence revealed that the gene contained three exons (a 5′ exon, an internal exon, and a 3′ exon) interrupted by two introns, which had a consensus GT-AG splice junction. Further analysis showed that the three exons corresponded to the signal peptide, mature peptide, and 3′-untranslated region (UTR), which are basic structural and functional domains of the LmKTT-1a precursor (Fig. 2B).

### Pharmacological Activities of rLmKTT-1a

**Figure 3:**
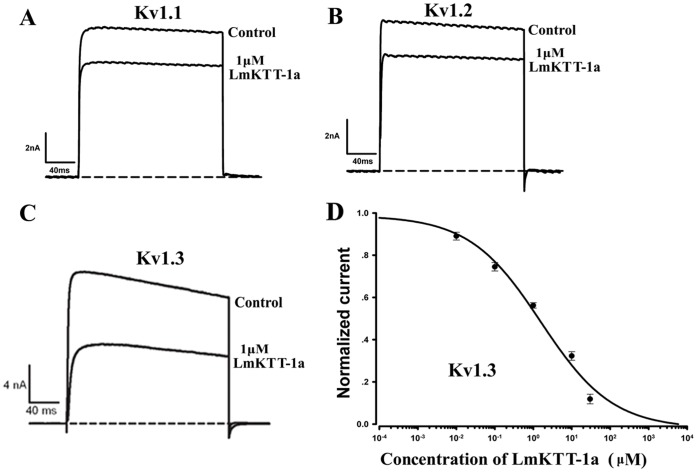
Inhibition of Kv1 potassium channel activity by LmKTT-1a. (A) Current traces of the Kv1.1 channel in the absence (control) or presence of 1 µM LmKTT-1a. (B) Current traces of the Kv1.2 channel in the absence (control) or presence of 1 µM LmKTT-1a. (C) Current traces of the Kv1.3 channel in the absence (control) or presence of 1 µM LmKTT-1a. (D) Concentration-dependent inhibition of Kv1.3 channel currents by LmKTT-1a. Data represent the mean ± S.D. of at least three experiments.

The biological effects of rLmKTT-1a on the Kv1.3 channel and trypsin protease were examined. The inhibitory effects of 1 µM rLmKTT-1a on the Kv1.1 channel, Kv1.2 channel, and Kv1.3 channel are shown (Fig. 3A–C). The results showed that 1 µM rLmKTT-1a inhibited the Kv1.3 current by approximately 50% with less effect on the Kv1.1 and Kv1.2 channels. The IC_50_ value of rLmKTT-1a on Kv1.3 channels was 1.58±0.73 µM (Fig. 3D). In addition, LmKTT-1a inhibited trypsin with a Ki value of about 0.14 µM. These results showed that LmKTT-1a is a unique bifunctional scorpion toxin with weak selectivity towards Kv1.3 channels.

### Solution Structure of LmKTT-1a

**Figure 4:**
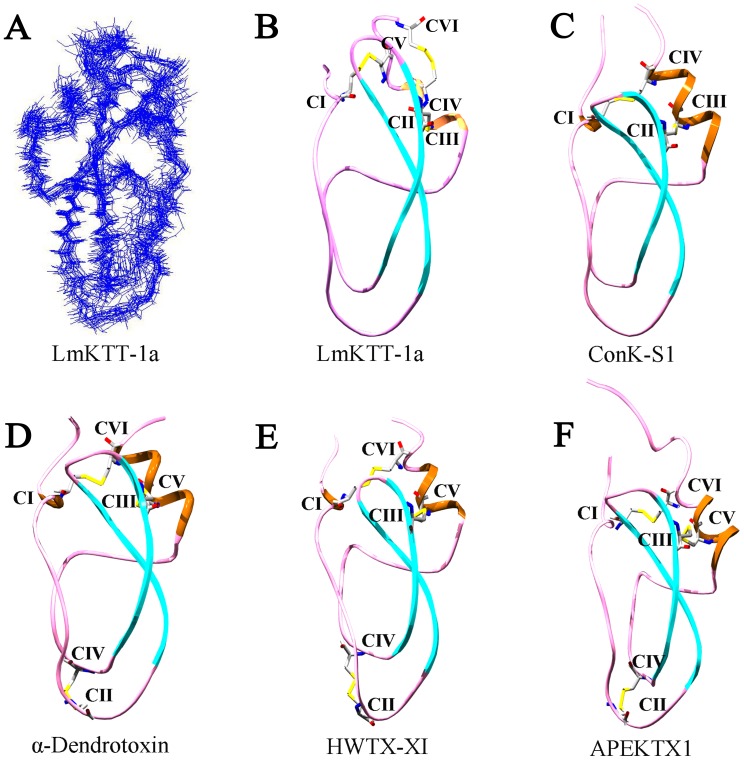
NMR solution structure of LmKTT-1a. A) Superposition of the 20 structures with lowest total energy. (B) Ribbon presentation of the backbone of LmKTT-1a from scorpion. (C) Ribbon presentation of the backbone of ConK-S1 from snail. (D) Ribbon presentation of the backbone of α-dendrotoxin from snake. (E) Ribbon presentation of the backbone of HWTX-XI from spider. (F) Ribbon presentation of the backbone of APEKTX1 from sea anemone.

Due to its novel cysteine patterns and genomic organization, the NMR structure of LmKTT-1a was determined. Multi-dimensional NMR spectra were obtained using ^13^C- and ^15^N-labeled LmKTT-1a, and the signals were fully assigned (Figs. S1 and S2). NMR structural statistics are summarized in Table 1. The structure of LmKTT-1a was determined using 709 NOE distance restraints, 30 dihedral angle restraints, and 26 H-bond restraints. A family of 20 accepted structures with the lowest energies and the best Ramachandran plots was selected to represent the three-dimensional solution structure of LmKTT-1a. The Ramachandran plot revealed that 86.5% of the residues were in the most favored regions, 10.9% were in additionally allowed regions, and 2.6% were in generously allowed regions. A diagram showing overlay between the backbone atoms of the 20 lowest energy structures of LmKTT-1a is presented in Fig. 4A. The root mean square deviation from the mean structure was 0.97+/−0.24 Å for the backbone and 1.56+/−0.30 Å for heavy atoms (Fig. 4A).

**Table 1:** Structural statistics for the family of 20 structures of LmKTT-1a.

Experimental constraints	
Intra-residue NOE (i = j)	426
Sequential NOE (|i-j| = 1)	163
Medium range (1<|i-j|<5)	41
Long range (|i-j|≥5)	79
Dihedral angle	30
Hydrogen bonds	26
Disulfide constraints	9
Target function^a^ (Å^2^)	3.15+/−0.63
RMSD Values^a^	
Average backbone RMSD to mean	0.97+/−0.24
Average heavy atom RMSD to mean	1.56+/−0.30
Ramachandran plot^b^	
Residues in most favored regions	86.5%
Residues in additional regions	10.9%
Residues in generously allowed regions	2.6%
Residues in disallowed regions	0.0%

a Data from Cyana.

b Data from PROCHECK-NMR.

The solution structure of LmKTT-1a resembled a typical Kunitz-type fold (Fig. 4B), which contains an N-terminal helix from Lys2 to Cys4, double-stranded anti-parallel β-sheets from Phe17 to Asn23 and Lys28 to Tyr34, and a C-terminal helix from Asp49 to Ala55. The C-terminal helix contained a unique disulfide link between Cys51 and Cys59, which was confirmed by their direct Nuclear Overhauser Enhancement (NOE) contacts and long-range NOEs of the adjacent residues (Fig. S4C). The structural integrity of LmKTT-1a was also maintained by two additional disulfide bridges located at Cys4–Cys54 and Cys29–Cys50 (Fig. S4A and B). The Cys4–Cys54 linkage shortened the distance between the N-terminal and C-terminal helices (Fig. S1). The β-sheet was stabilized by the connection between Cys29 and the C-terminal Cys50, which was the most rigid region of the structure.

The solution structure of LmKTT-1a was very similar to other Kunitz-type toxins, such as ConK-S1 from snail [29], α-dendrotoxin from snake [16], HWTX-XI from spider [26], and APEKTX1 from sea anemone [27] (Fig. 4). Although LmKTT-1a showed low amino acid sequence homologies with classical Kunitz-type peptides, it still adopted a conserved Kunitz-type fold. These findings suggest that peptides with a Kunitz-type fold maintain structural conservation yet show molecular diversity.

### Effects of the Unique Cys51–Cys59 Disulfide Bridge on Functions of LmKTT-1a

**Figure 5:**
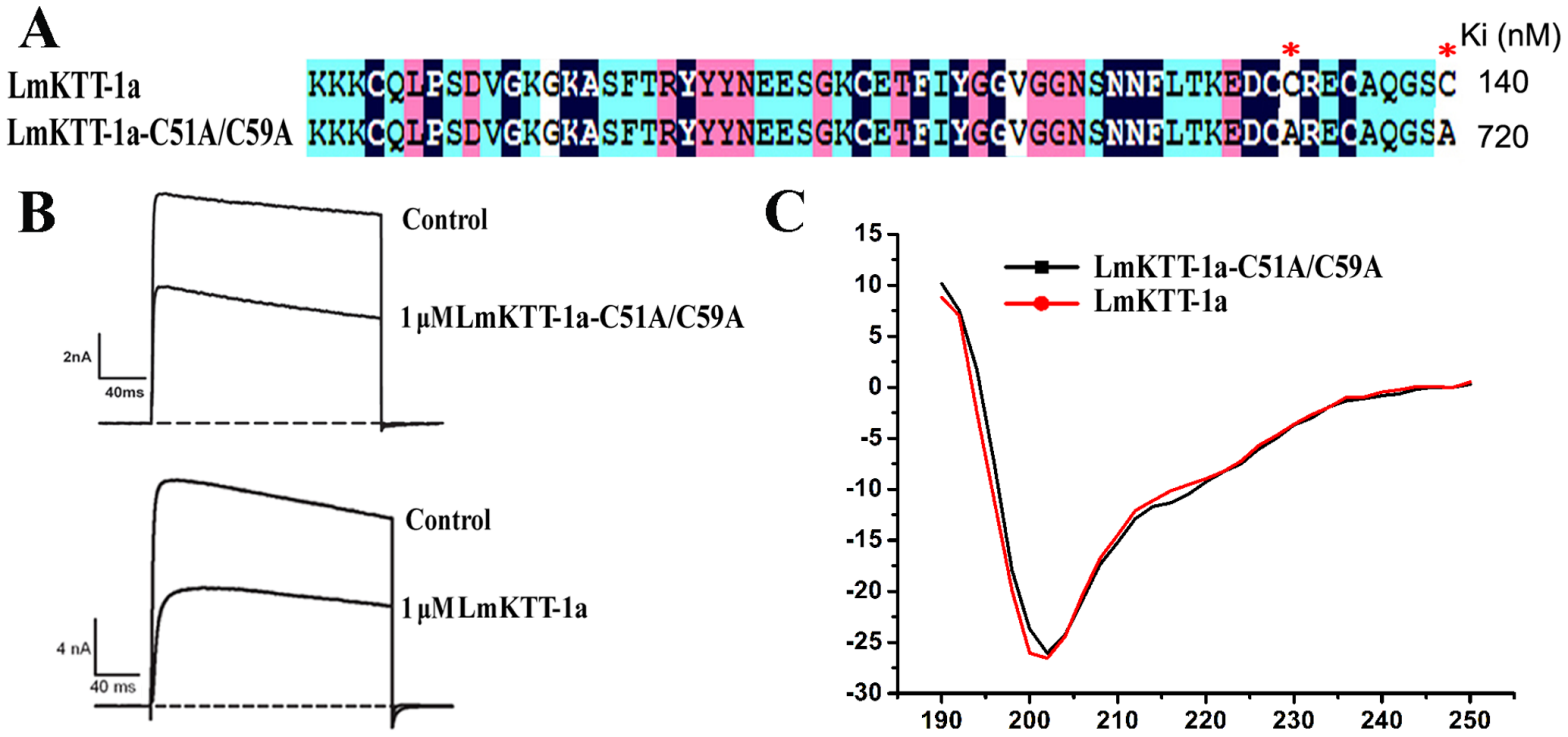
Functional evaluation of the unique disulfide bridge, Cys51–Cys59, in LmKTT-1a. ( A) A mutant LmKTT-1a-C51A/C59A lacking the unique disulfide bridge Cys51–Cys59 was designed from LmKTT-1a. Ki values for trypsin are labeled in bold font. (B) Current traces in the absence (control) or presence of 1 µM LmKTT-1a-C51A/C59A and LmKTT-1a. (C) Structural stability of the LmKTT-1a mutant, LmKTT-1a-C51A/C59A.

LmKTT-1a was a Kunitz-type toxin that adopted unique disulfide bridges (Figs. 1 and 4). To evaluate the function of characteristic disulfide bridges in LmKTT-1a, a mutant LmKTT-1a-C51A/C59A was designed (Fig. 5A), which had the same kind of disulfide bridge as the sea anemone ConK-S1 Kunitz-type toxin. Circular dichroism spectroscopy indicated that rLmKTT-1a-C51A/C59A had a secondary structure similar to LmKTT-1a (Fig. 5D). Enzyme and inhibitor reaction kinetics experiments showed that recombinant LmKTT-1a-C51A/C59A inhibited trypsin with 5-fold lower activity than wild-type LmKTT-1a (Fig. 5A). Electrophysiological experiments further demonstrated that LmKTT-1a-C51A/C59A inhibited the Kv1.3 channel to a similar degree as LmKTT-1a. Our results indicated that the unique Cys51–Cys59 disulfide bridge of LmKTT-1a had weak effects on its abilities to inhibit potassium channels and proteases, highlighting the evolutionary diversity and functional conservation of Kunitz-type toxins that have different disulfide bridge patterns.

## Discussion

**Figure 6:**
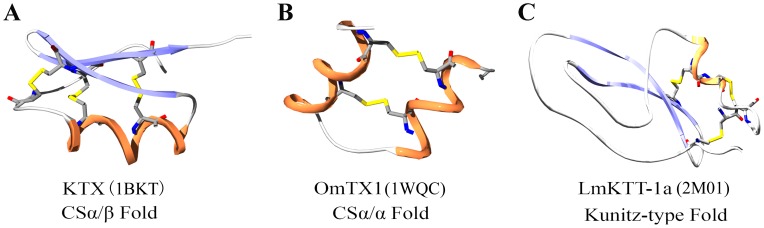
Diverse structural fold of scorpion potassium channel toxins. (A) Ribbon presentation of the backbone of KTX from the α-KTX subfamily, which has a CSα/β fold. (B) Ribbon presentation of the backbone of OmTx1 from the κ-KTX subfamily, which has a CSα/α fold. (C) Ribbon presentation of the backbone of LmKTT-1a from a new subfamily with a Kunitz-type fold.

Kunitz-type peptides are ubiquitous in numerous organisms including plants, animals, and microbes [30], [31]. The first Kunitz-type toxin to be reported was α-dendrotoxin, which was isolated from snake venom in 1974 [32]. Subsequently, additional Kunitz-type toxins were isolated from the venom glands of snakes, lizards, cone snails, spiders, and sea anemones [26]. Recently, scorpion Kunitz-type toxins were found and functionally characterized as trypsin and Kv1.3 channel inhibitors [13]. Based on previous findings, we determined the 3-D structure and genomic organization of a representative scorpion Kunitz-type toxin, LmKTT-1a, and discovered unique structural and evolutionary features of scorpion potassium channel toxins (Fig. 6). Our work highlights the third evolutionary ancestor of scorpion potassium channel toxins [33], [34].

### Diverse Genomic Organization of Scorpion Potassium Channel Toxins

**Figure 7:**
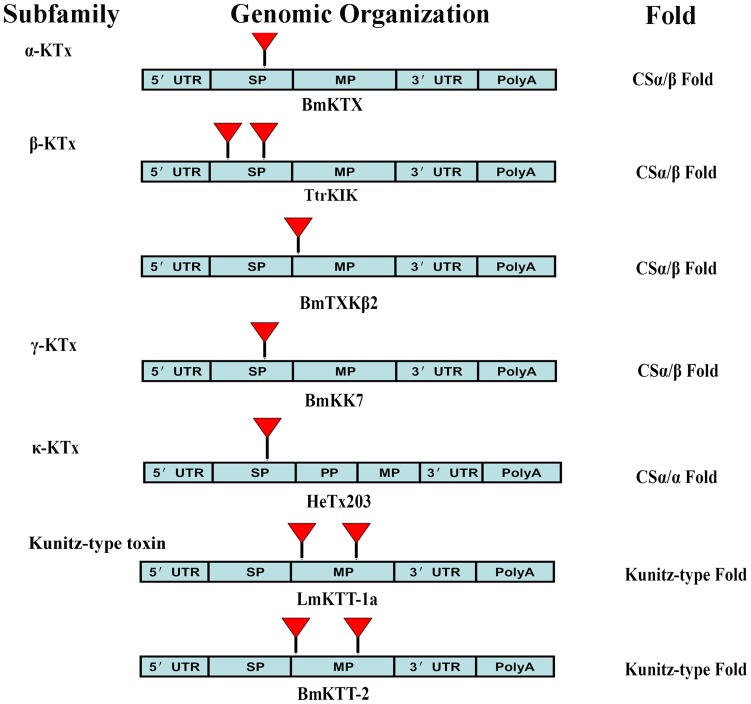
Comparison of the gene structures of representative scorpion potassium channel toxins. The gene structures of scorpion potassium channel toxins BmKTX from the α-KTX subfamily, which has a CSα/β fold, TtrKIK and BmTXKβ2 from the β-KTX subfamily, which has a CSα/β fold, BmKK7 from the γ-KTX subfamily, which has a CSα/β fold, HeTx203 from the κ-KTX subfamily, which has a CSα/α fold, and LmKTT-1a and BmKTT-2 from δ-KTX, the new subfamily of Kunitz-type fold toxins. The signal peptide (SP), propeptide (PP), mature peptide (MP), 5′-UTR, and 3′-UTR non-coding regions are shown. Introns are designated by triangles.

To compare the genomic organization of LmKTT-1a with known scorpion potassium channel toxins, we investigated the genomic organization of another scorpion Kunitz-type toxin, BmKTT-2, and representative scorpion potassium channel toxins from four known KTx subfamilies (Table 2 and Fig. 7). Our results showed that scorpion Kunitz-type potassium channel toxins have a unique genomic organization, which is different from that of scorpion toxins in the other four KTx subfamilies. The new genomic organization of scorpion Kunitz-type toxins further revealed the diversity of scorpion potassium channel toxins.

**Table 2:** Diversity of potassium channel toxins (KTxs) from scorpion venom.

Classification	Gene Structure	3-D Fold	Activity
α-KTx subfamily	One intron in the signal peptide	CSα/β	Kv1, hERG, SKCa, BKCa, IKCa effectors
β-KTx subfamily	One intron in the mature peptide or two introns in the signal peptide	CSα/β	Kv1.3 effectors, antimicrobial and anti-malaria
γ-KTx subfamily*	One intron in the signal peptide	CSα/β	hERG effectors
κ-KTx subfamily*	One big intron in the signal peptide	CSα/α	Kv1.3 effectors
δ-KTx subfamily*	Two introns in the mature peptide	Kunitz-type	Kv1.3 effectors and Trypsin inhibitors

* The genomic organization of this subfamily was reported firstly in this work.

### Structural Diversity of Scorpion Potassium Channel Toxins

The only structural folds that have been identified in scorpion potassium channel toxins are CS-α/β (cysteine-stabilized α-helix and β sheets) [35] and CS-α/α (cysteine-stabilized α-helix-loop-helix) [5]. Scorpion toxin LmKTT-1a possessed a unique cysteine framework and adopted a conserved Kunitz-type fold. To our knowledge, the LmKTT-1a structure is the third structural fold to be discovered for scorpion potassium channel toxins [15], demonstrating their structural diversity (Fig. 6).

### A Unified Nomenclature for Scorpion Kunitz-type Toxins: δ-KTx Subfamily

**Figure 8:**
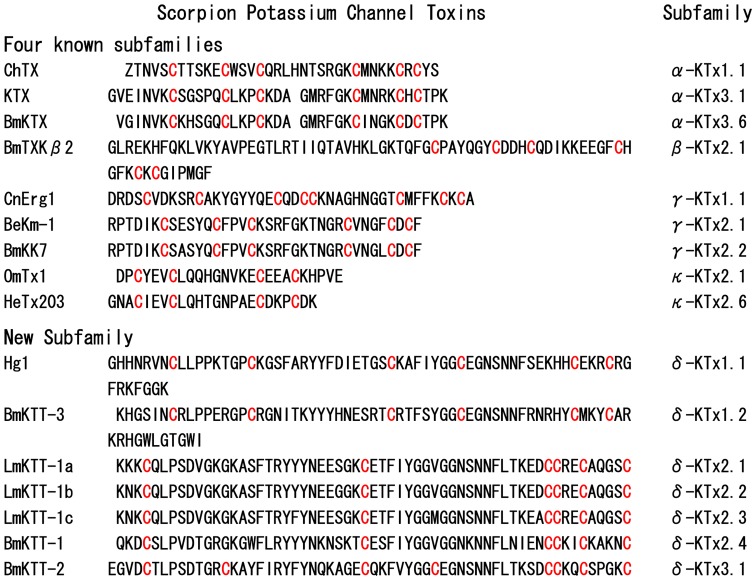
Molecular diversity and classification of scorpion potassium channel toxins. Representative potassium channel toxins from the α-KTxs, β-KTxs, γ-KTxs, κ-KTxs, and δ-KTxs subfamily are listed. All members from the δ-KTxs subfamily with a Kunitz-type fold are shown.

Currently, the principle of classification and unified nomenclature for scorpion potassium channel toxins is based on their amino acid sequence similarities, type of structural folds, and genomic organization [5], [6]. Based on the structural, functional, and genomic comparison of scorpion Kunitz-type toxins with toxins from the four known subfamilies, we propose to classify scorpion Kunitz-type potassium channel toxins as a new KTx subfamily called δ-KTx. To our knowledge, this new KTx subfamily from scorpion contains seven members and can be classified into three groups with three different disulfide bridge patterns (Fig. 8).

### Evolutionary Diversity of Scorpion Potassium Channel Toxins

**Figure 9:**
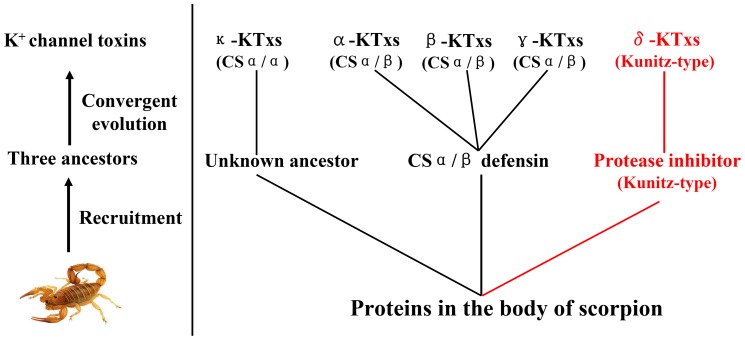
Schematic diagram of the evolution of scorpion potassium channel toxins. Three putative ancestors were recruited from scorpion proteins to generate diverse potassium channel toxins from five different subfamilies (α-KTxs, β-KTxs, γ-KTxs, κ-KTxs, and δ-KTxs) with three different structural folds (CSα/β, CSα/β, and Kunitz-type).

Given the selective pressure that occurs during the course of evolution, conservation between two proteins or peptides at the levels of genomic organization and 3-D structure provides strong evidence for a common evolutionary origin [36]. Previous research has shown that scorpion potassium channel toxins with a CSα/β fold share a common ancestor [34]. In this work, we provide the first report of the genomic organization of scorpion potassium channel toxins with Kunitz-type and CSα/α structural folds (Figs. 2, S5, and S6). Our results showed that scorpion potassium channel toxins with Kunitz-type fold and CSα/α fold adopted their unique genomic organization and structural folds and are completely different from the classical CSα/β toxins (Figs. 7 and S7). Combining the genomic organization, 3-D fold, and functional data together suggested that scorpion potassium channel toxins might have evolved from three different ancestors (Fig. 9). Our results highlight a new role for convergent evolution of animal toxins and demonstrate the evolutionary diversity of scorpion potassium channel toxins.

### Conclusions

In summary, we present the first characterization of the NMR solution structure and genomic organization of scorpion Kunitz-type potassium channel toxin. LmKTT-1a possessed a novel genomic organization and a conserved Kunitz-type structural fold with one α-helix and two β-sheets. Genomic and structural features were different from that of the classical scorpion potassium channel toxins, such as α-KTxs, β-KTxs, γ-KTxs, and κ-KTxs. Based on these analyses, we propose that a new subfamily of scorpion potassium channel toxins exists, which we have named δ-KTx. Our results highlight the structural, genomic, and evolutionary diversity of scorpion potassium channel toxins. These results may accelerate the design and development of diagnostic and therapeutic peptide agents for human potassium channelopathies.

## Supporting Information

Figure S1 **^1^H-^15^N HSQC spectrum of LmKTT-1a.** The spectrum was labeled with all of the assignments including the expression tag (from residue tG1 to tM20) and the intact peptide (from residue K1 to C59). The amino acid sequence of rLmKTT-1a is GSSHHHHHHSSGLVPRGSHMKKKCQLPSDVGKGKASFTRYYYNEESGKCETFIYGGVGGNSNNFLTKEDCCRECAQGSC. The amide protons of Histidine residues (tH4–tH8) from the His-tag are severely overlapped and were not assigned. All assigned cross-peaks have been labeled with a one-letter amino acid code.
(TIF)

Figure S2 **NOESY spectra of LmKTT-1a.** Sequential assignment strips of the intact peptide are shown by 3D ^15^N-^1^H NOESY-HSQC.
(TIF)

Figure S3 **^1^H-^15^N HSQC spectrum of LmKTT-1a after dissolving it in D_2_O for 8 hours.**
(TIF)

Figure S4 **NOE evidences for disulfide bonds C4–C54 (A), C29–C50 (B) and C51–C59 (C).** The NOE connections were illustrated by ^13^C-edited NOESY-HSQC spectrum and a stick representation of the structure. The structure in the right panel was produced using the MOLMOL program. The sidechains of relevant residues were shown in red and indicated by residue name and number.
(TIF)

Figure S5 **The HeTx203 gene sequence from the κ-KTx subfamily, which has a CSα/α fold, is shown.** The signal peptide sequence predicted from the nucleotide sequence is underlined.
(TIF)

Figure S6 **The BmKTT-2 gene sequence from the δ-KTx subfamily, which has a Kunitz-type fold, is shown.** The signal peptide sequence predicted from the nucleotide sequence is underlined.
(TIF)

Figure S7 **The BmKK7 gene sequence from the γ-KTx subfamily, which has a CSα/β fold, is shown.** The signal peptide sequence predicted from the nucleotide sequence is underlined.
(TIF)
