# Supplementary figures and images for: Genomic and Structural Characterization of Kunitz-Type Peptide LmKTT-1a Highlights Diversity and Evolution of Scorpion Potassium Channel Toxins
Source: PLoS One. 2013 Apr 3;8(4):e60201. doi: 10.1371/journal.pone.0060201 (PMC3616063; doi:10.1371/journal.pone.0060201)

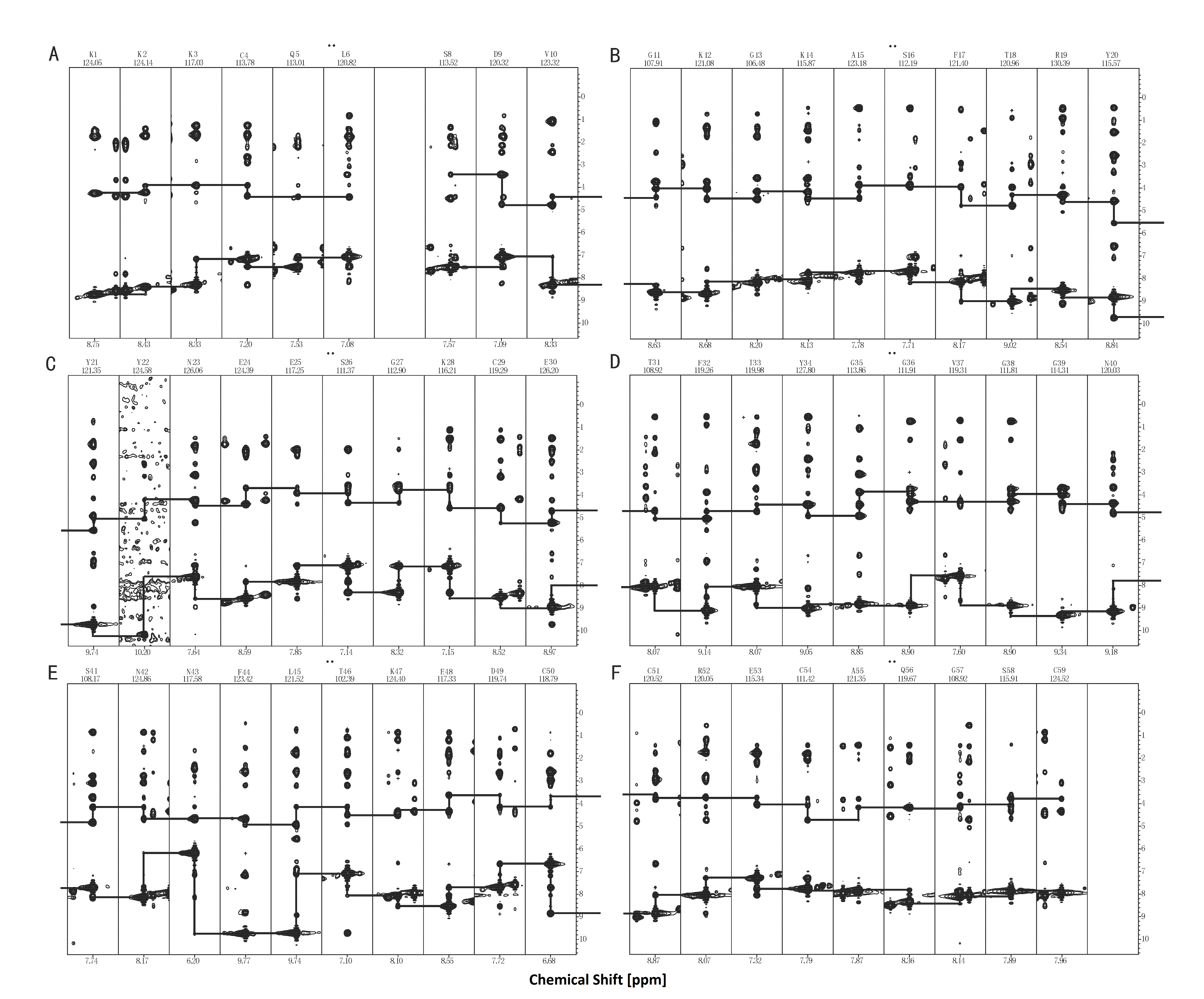

Supplement: Figure S2 — NOESY spectra of LmKTT-1a. Sequential assignment strips of the intact peptide are shown by 3D 15N-1H NOESY-HSQC. (TIF) [file pone.0060201.s002.tif]

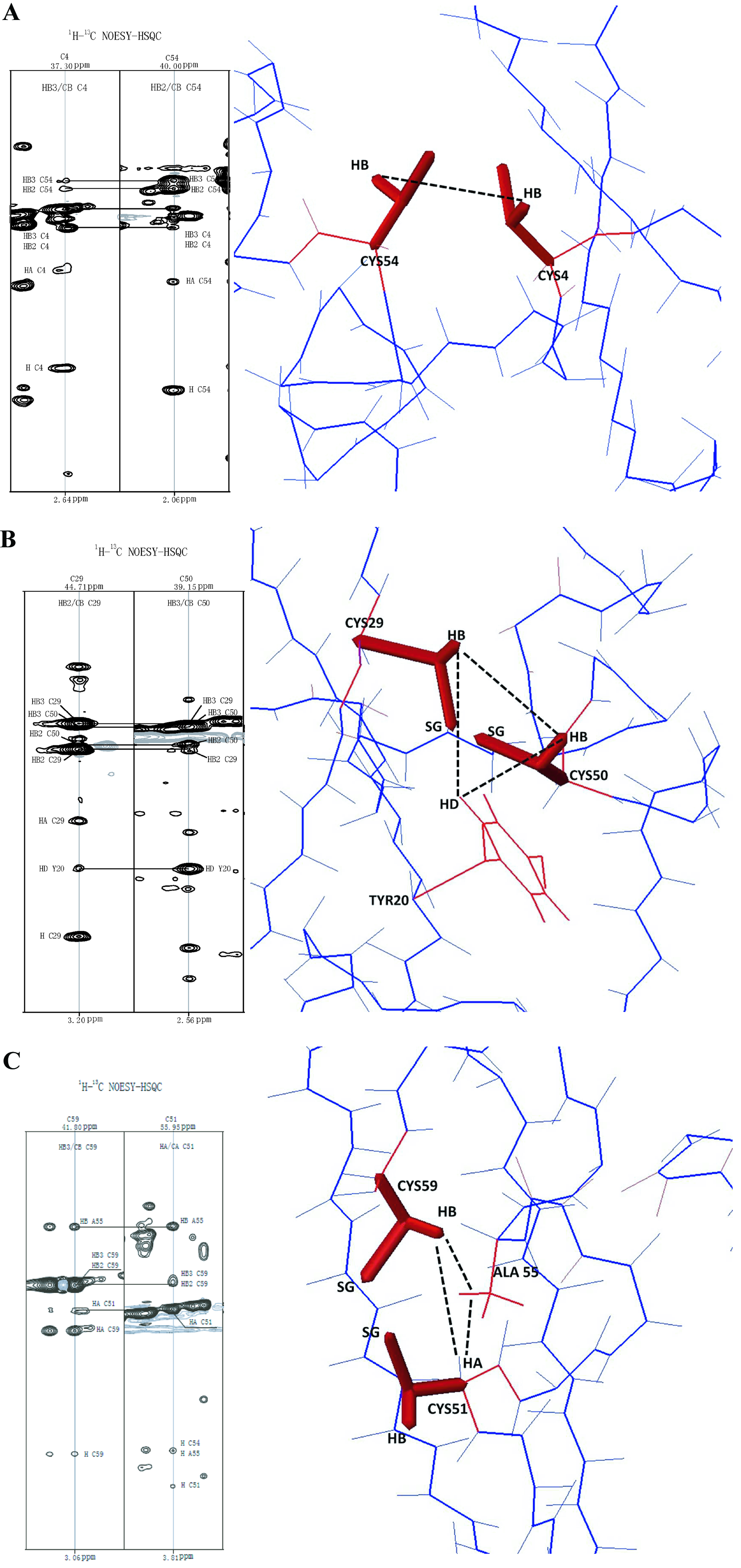

Supplement: Figure S4 — NOE evidences for disulfide bonds C4–C54 (A), C29–C50 (B) and C51–C59 (C). The NOE connections were illustrated by 13C-edited NOESY-HSQC spectrum and a stick representation of the structure. The structure in the right panel was produced using the MOLMOL program. The sidechains of relevant residues were shown in red and indicated by residue name and number. (TIF) [file pone.0060201.s004.tif]

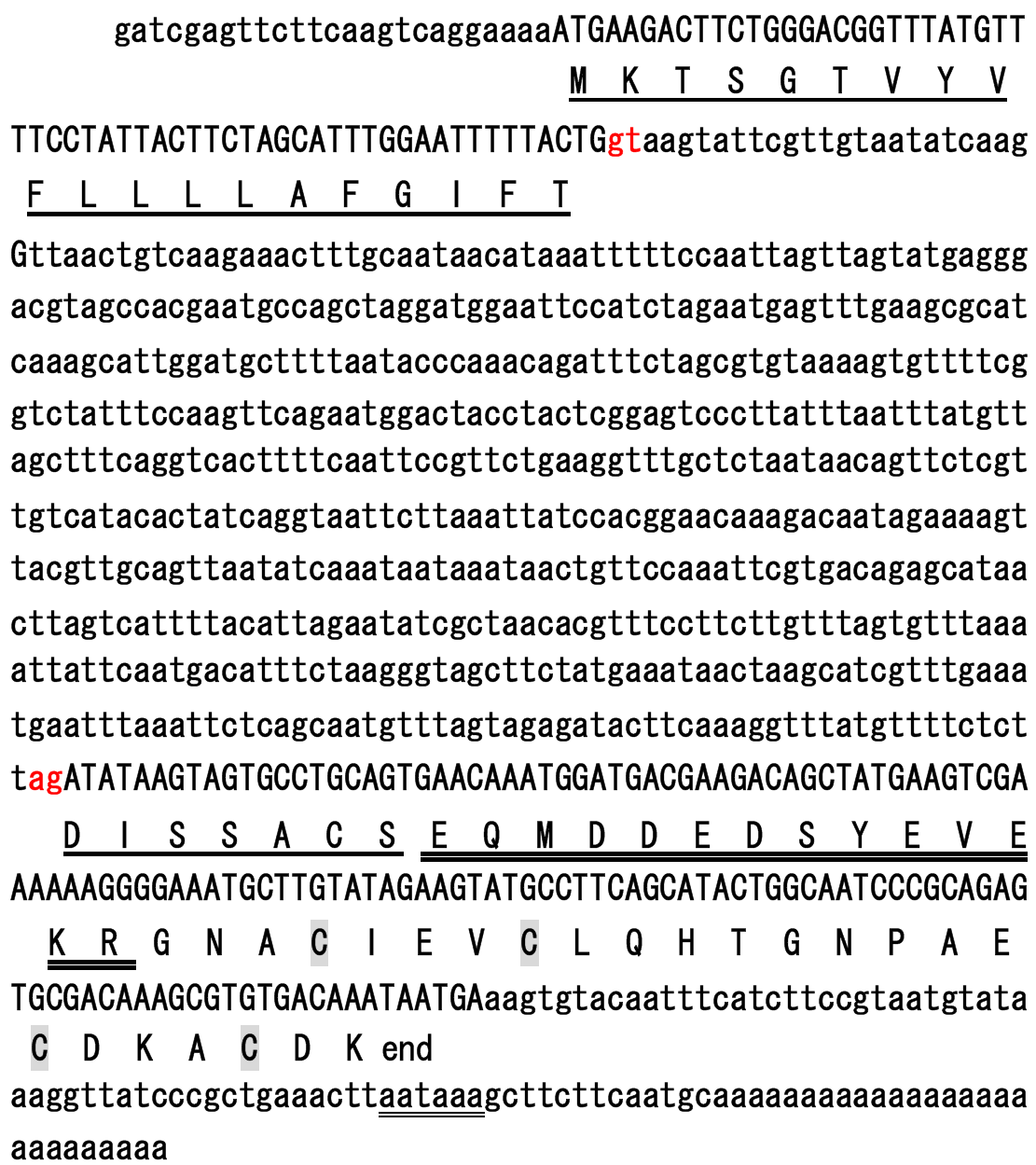

Supplement: Figure S5 — The HeTx203 gene sequence from the κ-KTx subfamily, which has a CSα/α fold, is shown. The signal peptide sequence predicted from the nucleotide sequence is underlined. (TIF) [file pone.0060201.s005.tif]

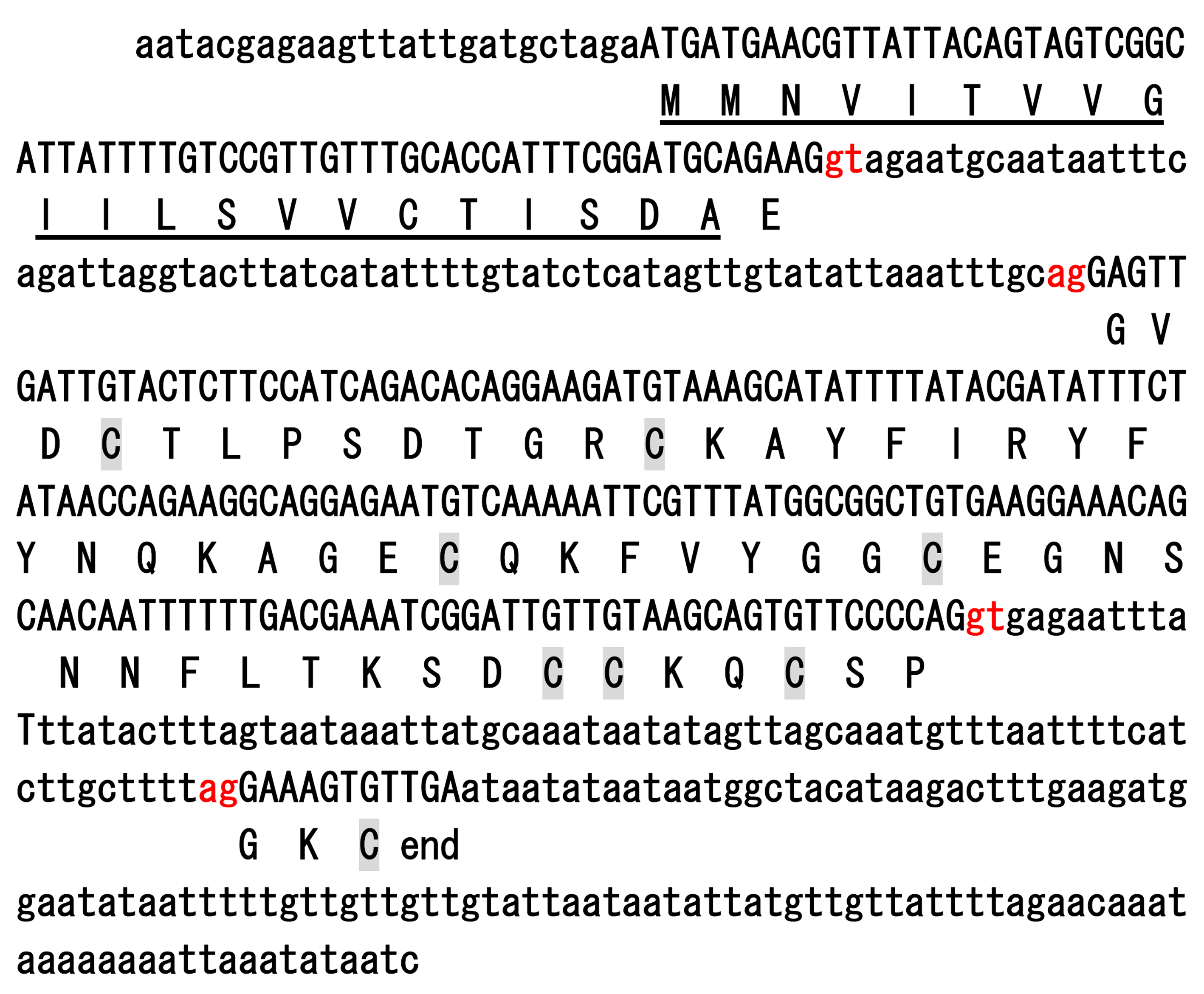

Supplement: Figure S6 — The BmKTT-2 gene sequence from the δ-KTx subfamily, which has a Kunitz-type fold, is shown. The signal peptide sequence predicted from the nucleotide sequence is underlined. (TIF) [file pone.0060201.s006.tif]

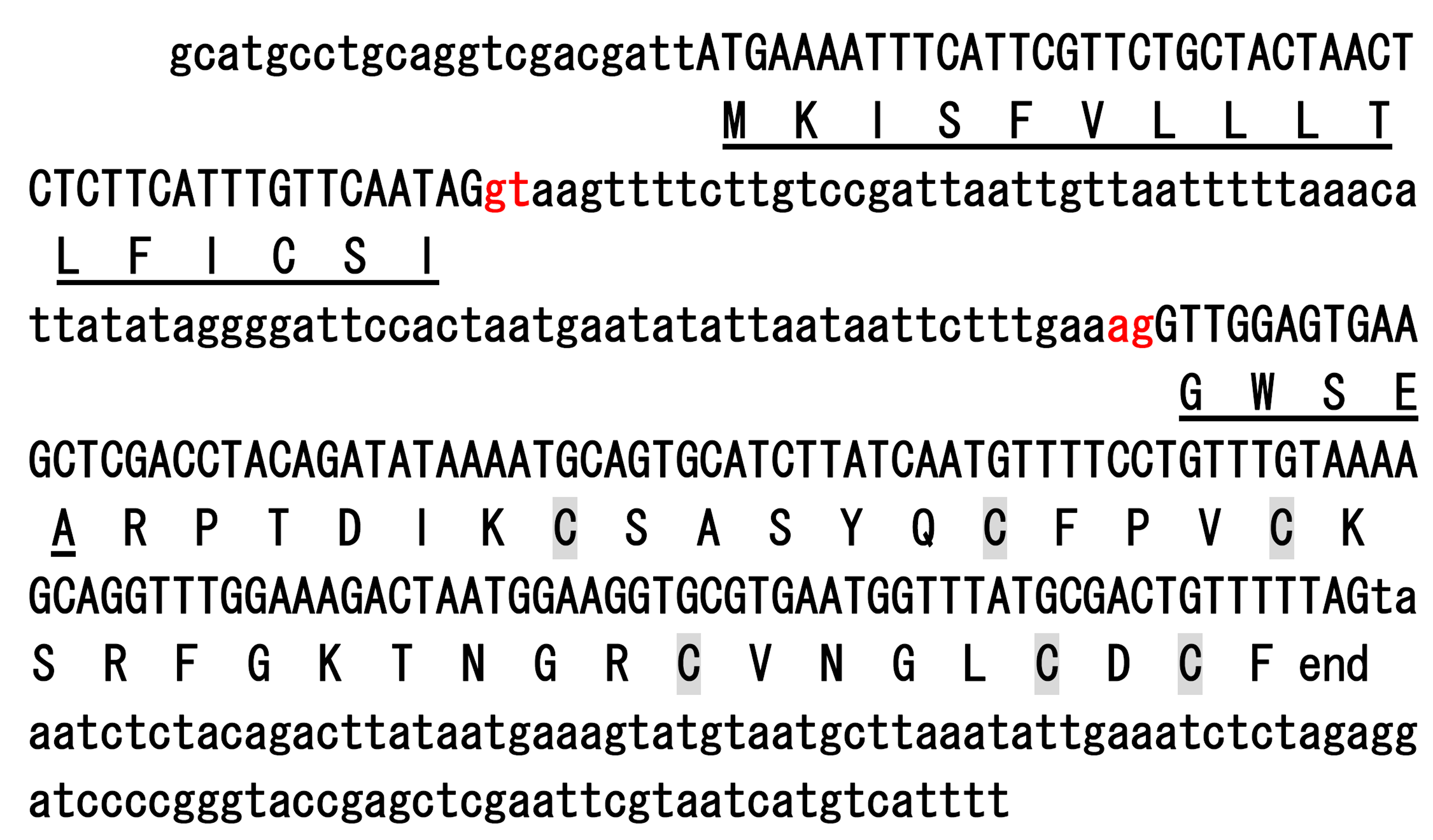

Supplement: Figure S7 — The BmKK7 gene sequence from the γ-KTx subfamily, which has a CSα/β fold, is shown. The signal peptide sequence predicted from the nucleotide sequence is underlined. (TIF) [file pone.0060201.s007.tif]
